# Association Between Early Childhood Caries and Systemic Inflammatory Profiles: A Retrospective Analysis of Children Undergoing Dental Treatment Under General Anesthesia

**DOI:** 10.3390/children13050691

**Published:** 2026-05-19

**Authors:** Banu Çiçek Tez Yaşar, Akif Burak Çakmak, Hacer Eberliköse, Arif Yiğit Güler, Bahar Başak Kızıltan Eliaçık, Hakan Alpay Karasu

**Affiliations:** 1 Department of Pediatric Dentistry, Faculty of Dentistry, Ankara Medipol University, 06570 Ankara, Türkiye; banu.tez@ankaramedipol.edu.tr; 2 Department of General Anesthesia and Reanimation, Ankara Medical Park Hospital, 06570 Ankara, Türkiye; 3 Department of Oral and Maxillofacial Surgery, Faculty of Dentistry, Ankara Medipol University, 06570 Ankara, Türkiye; arif.guler@ankaramedipol.edu.tr (A.Y.G.); hakan.karasu@ankaramedipol.edu.tr (H.A.K.); 4 Department of Pediatric Dentistry, Hamidiye Faculty of Dentistry, University of Health Sciences, 34668 İstanbul, Türkiye; basak.eliacik@sbu.edu.tr

**Keywords:** early childhood caries, systemic inflammation, neutrophil-to-lymphocyte ratio, systemic immune-inflammation index, complete blood count, inflammatory biomarkers

## Abstract

**Highlights:**

**What are the main findings?**

- A non-linear pattern was observed between ECC severity and systemic inflammatory markers, with higher inflammatory activity in the moderate dmft group.
- Pairwise analyses revealed higher NLR and SII levels in the moderate dmft group compared to the high severity group.

**What is the implication of the main finding?**

- Systemic inflammatory responses in ECC may be more strongly influenced by disease-specific characteristics than by cumulative caries burden.
- CBC-derived indices such as NLR and SII may serve as accessible but supportive markers in pediatric dental assessment.

**Abstract:**

Background/Objectives: Early childhood caries (ECC) is a chronic inflammatory condition that may impose a systemic burden in pediatric patients. This study aimed to evaluate the association between dental caries severity, classified by dmft (decayed, missing, and filled teeth for primary dentition) scores, and preoperative systemic inflammatory markers derived from routine complete blood counts (CBC). Methods: This retrospective study included 159 children aged 36–71 months. Participants were categorized into three groups based on dmft scores: low (0–3), medium (4–8), and high (≥9). Hematological parameters and inflammatory indices, including neutrophil-to-lymphocyte ratio (NLR) and systemic immune-inflammation index (SII), were analyzed using one-way ANOVA with post hoc comparisons. Results: Significant differences were observed among dmft groups for neutrophil and lymphocyte percentages (*p* = 0.026 and *p* = 0.027) and lymphocyte count (*p* = 0.020). The medium severity group demonstrated higher neutrophil levels and lower lymphocyte values compared to the high severity group (*p* < 0.05). Although overall group differences for NLR and SII were not statistically significant (*p* > 0.05), both markers were significantly higher in the medium group than in the high group (*p* < 0.05). No significant differences were found in hemoglobin, RDW, or platelet parameters. Conclusions: A non-linear trend was observed, with relatively elevated inflammatory markers in the moderate dmft group. These findings suggest that systemic inflammation in ECC is more closely related to disease characteristics than to caries burden alone. CBC-derived parameters may provide supportive but limited value for assessing systemic inflammatory status in pediatric dental patients.

## 1. Introduction

Early childhood caries (ECC) is defined as the presence of one or more decayed (non-cavitated or cavitated lesions), missing (due to caries), or filled tooth surfaces in any primary tooth in a child aged 71 months or younger [1,2]. As the most common chronic infectious disease of childhood, ECC remains a significant global public health concern, with prevalence rates reported as high as 67.3% in some preschool populations. The etiology of the disease is multifactorial, resulting from a complex interaction between cariogenic microorganisms, a diet rich in fermentable carbohydrates, and host susceptibility [3,4].

Despite advances in preventive dentistry, ECC continues to disproportionately affect preschool-aged children and is frequently associated with pain, infection, impaired nutrition, and reduced quality of life. Importantly, in severe cases, comprehensive dental rehabilitation under general anaesthesia (GA) becomes necessary, reflecting extensive dental treatment needs and high caries burden in this patient population [5].

While ECC is traditionally considered a localized biofilm-driven disease, inflammatory and immune responses associated with active odontogenic infection may have effects beyond the oral cavity [6]. The concept of oral–systemic interaction has been well established in periodontal diseases; however, the systemic implications of dental caries—particularly in early childhood—remain insufficiently explored. Current evidence suggests that dental caries may contribute to systemic inflammatory responses via host–microbiome interactions, yet the clinical relevance of this relationship is still not fully understood.

From a pathophysiological perspective, ECC involves sustained activation of the host immune response against cariogenic microorganisms. Neutrophils, as key effectors of innate immunity, play a central role in this process and contribute to both local tissue responses and systemic inflammatory signaling. Chronic exposure to odontogenic infection may therefore induce measurable alterations in circulating hematological parameters, reflecting a systemic inflammatory state [7,8].

In clinical practice, the assessment of systemic inflammation has traditionally relied on markers such as C-reactive protein (CRP) [9]. However, in recent years, indices derived from routine complete blood counts (CBC) have emerged as inexpensive, easily accessible, and reliable biomarkers for evaluating the immune-inflammatory balance. Specifically, next-generation markers such as the Neutrophil-to-Lymphocyte Ratio (NLR) and the Systemic Immune-Inflammation Index (SII)—which integrates neutrophil, platelet, and lymphocyte counts—have demonstrated strong diagnostic and prognostic value in various systemic diseases, including cardiovascular disorders, malignancies, and acute infections [10,11,12].

Children undergoing dental treatment under general anesthesia represent a clinically distinct subgroup characterized by extensive dental treatment needs and high caries burden. Although preoperative hematological data are routinely available in this population, the relationship between ECC severity and systemic hematological alterations has not been clearly defined. Furthermore, whether systemic inflammatory responses increase proportionally with caries burden or follow a more complex pattern remains unclear. Therefore, the present study aimed to evaluate the association between early childhood caries and preoperative hematological profiles in children undergoing dental treatment under general anesthesia. Given the limited and inconsistent evidence in this field, this study specifically sought to explore the nature and pattern of the relationship between caries severity and systemic inflammatory markers derived from routine CBC parameters, rather than assuming a linear relationship. A better understanding of this association may provide new insights into the possible inflammatory implications of ECC and support the potential use of hematological indices as adjunctive tools in pediatric dental risk assessment.

## 2. Materials and Methods

### 2.1. Study Design and Ethical Approval

The study was conducted in accordance with the Declaration of Helsinki and approved by the Non-Interventional Clinical Research Ethics Committee of Ankara Medipol University (protocol code: 55, approval date: 3 March 2026). Due to the retrospective design of the research, no additional clinical intervention, supplementary laboratory tests, or extra biological sampling were performed on the participants specifically for this study.

All data were extracted from existing clinical and laboratory records generated during routine dental examinations and preoperative assessments. To ensure patient privacy, all personal identifiers were removed, and the data were anonymized prior to statistical analysis. Since the research relied on the retrospective evaluation of records obtained during standard clinical practice, and a general institutional consent—which explicitly permits the use of anonymized clinical data, radiographs, and documents for scientific and educational purposes—was obtained from the legal guardians of all patients at the time of their initial admission, the requirement for additional informed consent was waived.

### 2.2. Study Population and Selection Criteria

The study population consisted of preschool children aged 36–71 months who underwent dental treatment under general anesthesia between 1 January 2023, and 1 December 2025. A total of 159 patients (78 females, 81 males) were included in the final analysis.

#### 2.2.1. Inclusion Criteria

(1) Patients aged 3–6 years; (2) scheduled for dental treatment under general anesthesia; (3) classified as ASA I (systemically healthy); (4) complete dmft index records; and (5) available preoperative Complete Blood Count (CBC) results.

#### 2.2.2. Exclusion Criteria 

(1) Chronic systemic or hematological diseases (ASA II and above); (2) acute infection or fever during the preoperative period; (3) use of antibiotics, corticosteroids, or immunosuppressive drugs within one month prior to blood sampling; and (4) incomplete or contradictory clinical records.

### 2.3. Sample Size Calculation

Sample size was determined using G*Power 3.1.9.7 software (Heinrich Heine University Düsseldorf, Düsseldorf, Germany). Based on a reference study by Saraç et al. (2025), considering NLR as the primary outcome variable with an effect size (Cohen’s d) of 0.413, an alpha level of 0.05, and 0.80 power, a minimum of 148 patients was required [13]. The final sample size of 159 exceeded this requirement.

### 2.4. Dental Evaluation and Severity Grouping

Dental caries status was assessed using the dmft index (decayed, missing, and filled teeth for primary dentition) following World Health Organization (WHO) criteria. To analyze the relationship between caries severity and systemic inflammation, patients were categorized into three groups based on their total dmft scores:

- Low Severity Group (*n* = 11): dmft scores between 0 and 3.
- Medium Severity Group (*n* = 92): dmft scores between 4 and 8.
- High Severity Group (*n* = 56): dmft scores 9 and above.

### 2.5. Hematological Parameters and Indices

Preoperative CBC data were retrospectively extracted from laboratory records. The parameters recorded included Neutrophil count (NEU#), Lymphocyte count (LYM#), Platelet count (PLT), and Red Cell Distribution Width parameters (RDW-CV and RDW-SD). Based on these values, the following systemic inflammatory indices were calculated:

- Neutrophil-to-Lymphocyte Ratio (NLR): NEU#/LYM#.
- Platelet-to-Lymphocyte Ratio (PLR): PLT/LYM#.
- Systemic Immune-Inflammation Index (SII): (NEU# × PLT)/LYM# [14].

### 2.6. Statistical Analysis

Statistical analysis was performed using IBM SPSS Statistics version 21.0 (IBM Corp., Armonk, NY, USA). Descriptive statistics were presented as mean ± standard deviation (SD) and 95% confidence intervals, or frequencies where appropriate. Normality of data distribution was assessed using the Shapiro–Wilk test prior to intergroup comparisons. Since the analyzed variables demonstrated approximately normal distributions, parametric tests were preferred. One-way analysis of variance (ANOVA) was used to compare continuous variables among the three independent dmft severity groups, followed by Tukey’s post hoc test for pairwise comparisons. Tukey’s test was preferred because it allows reliable multiple comparisons while reducing the risk of type I error associated with multiple testing. Categorical variables were analyzed using the chi-square test. A *p*-value < 0.05 was considered statistically significant.

## 3. Results

A total of 159 patients were included in the study, comprising 78 females (49.1%) and 81 males (50.9%). The mean age of the participants was 4.45 ± 1.46 years (Table 1). Based on their dmft scores, the patients were categorized into three groups: low (*n* = 11), medium (*n* = 92), and high (*n* = 56). Significant differences were observed across the dmft groups for several hematological parameters (Table 2).

**Figure 1:**
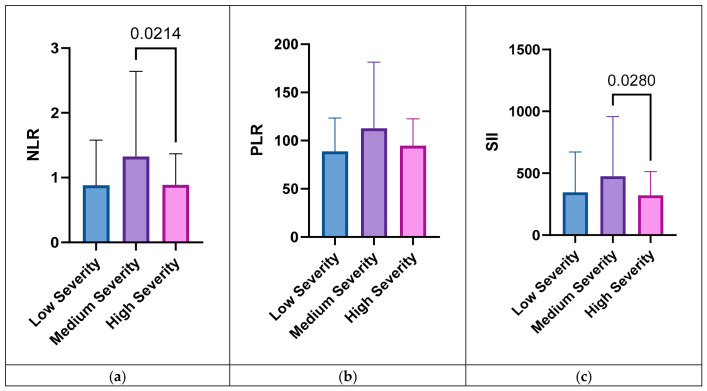
Distribution of systemic inflammatory indices across dmft severity groups. (**a**) Neutrophil-to-lymphocyte ratio (NLR), (**b**) platelet-to-lymphocyte ratio (PLR), and (**c**) systemic immune-inflammation index (SII) in low-, medium-, and high-severity dmft groups. Data are presented as mean ± SD. Pairwise comparisons indicated higher NLR and SII values in the medium severity group compared to the high severity group (*p* < 0.05), while overall group differences were not statistically significant.

Neutrophil percentage (NEUT%) differed significantly among groups (*p* = 0.026), with post hoc analysis indicating higher values in the medium group compared to the high group (*p* = 0.002). Similarly, lymphocyte percentage (LYM%) showed significant variation (*p* = 0.027), with lower values in the medium group compared to the high group (*p* < 0.001). The absolute lymphocyte count (LYM#) also differed significantly between groups (*p* = 0.020), particularly between the medium and high groups (*p* = 0.036) (Figure 1).

Regarding systemic inflammatory indices, no statistically significant differences were found among groups for NLR (*p* = 0.273) or SII (*p* = 0.388) in overall comparisons. However, post hoc analyses revealed significantly higher NLR and SII values in the medium group compared to the high group (*p* = 0.021 and *p* = 0.028, respectively). No significant differences were observed in platelet count (PLT), red cell distribution width parameters (RDW-CV and RDW-SD), or hemoglobin (Hb) levels across the groups (*p* > 0.05).

## 4. Discussion

The present study evaluated the relationship between early childhood caries (ECC) severity and systemic inflammatory status using CBC-derived parameters in children undergoing dental treatment under general anesthesia. The findings demonstrated a non-linear trend in the distribution of inflammatory markers, with relatively higher NLR and SII values observed in the moderate dmft group. However, no statistically significant differences were identified in overall group comparisons for these indices. These results suggest that systemic inflammatory responses in ECC may not increase proportionally with caries burden and may instead vary according to disease characteristics beyond numerical severity. The systemic implications of dental caries remain a subject of ongoing investigation. Although previous studies have proposed potential links between ECC and systemic inflammatory conditions, current evidence is still insufficient to establish definitive causal relationships. Nevertheless, persistent microbial challenge and host immune activation associated with ECC may contribute to low-grade systemic inflammation [5].

Neutrophils play a central role in innate immunity and represent the first line of defense against microbial invasion. An increase in neutrophil activity accompanied by relative lymphocyte suppression is widely recognized as an indicator of systemic inflammation and is reflected in elevated NLR values [15]. In the present study, significantly higher neutrophil percentages and lower lymphocyte levels were observed in the moderate dmft group compared to the high severity group, supporting the presence of an altered inflammatory profile in this subgroup. Previous studies further support the inflammatory dimension of ECC. Elevated levels of pro-inflammatory cytokines such as IL-6, IL-8, and TNF-α have been reported in children with ECC, indicating systemic immune activation beyond the oral cavity [16]. Similarly, untreated dental caries has been associated with odontogenic infections capable of inducing both local and systemic inflammatory responses [13]. In addition, systemic inflammatory indices such as SII have been shown to reflect disease severity in odontogenic infections, while NLR has been widely validated as a marker of inflammatory status across various oral and systemic conditions [17,18].

The absence of significant differences in hemoglobin, RDW, and platelet parameters suggests that ECC-related inflammation may remain subclinical and predominantly reflected in leukocyte dynamics rather than erythrocyte indices. In pediatric populations, inflammatory responses are often more evident in leukocyte profiles, while platelet changes may vary depending on the nature and duration of inflammation [19]. It is also important to consider that all participants in this study were children undergoing dental treatment under general anesthesia, representing a subgroup with extensive dental treatment needs and severe ECC experience. Previous studies have shown that such patients typically present with severe ECC and extensive treatment needs [20]. The variability observed in inflammatory markers within this relatively homogeneous population further supports the notion that caries severity alone may not fully explain systemic inflammatory responses.

The observed pattern in this study may be partially explained by the inherent limitations of the dmft index. Although dmft is widely used to quantify caries experience, it does not directly reflect current inflammatory activity, lesion depth, pulpal involvement, or the biological severity of active odontogenic infection. Therefore, teeth included within the dmft index may represent heterogeneous clinical conditions with substantially different inflammatory potential. For example, previously restored or extracted teeth may not constitute active inflammatory foci, whereas a limited number of biologically active lesions may induce a stronger systemic immune response. This limitation may partially explain the non-linear inflammatory pattern observed in the present study. As a result, similar or even higher dmft scores may represent heterogeneous clinical conditions. This limitation has been well recognized in the literature and may influence the interpretation of disease severity in relation to biological outcomes [2,21]. It is therefore plausible that moderate dmft values may correspond to cases with relatively more active or advanced lesions, potentially eliciting stronger inflammatory responses compared to higher dmft scores reflecting a greater number of lesions without equivalent biological activity. This finding may further suggest that active odontogenic infection and ongoing inflammatory activity could represent stronger triggers for systemic immune responses than the chronic presence of multiple carious lesions alone. Therefore, systemic inflammatory burden in ECC may be more closely associated with biological activity and lesion progression rather than cumulative dmft scores alone. However, this interpretation should be considered cautiously and requires further validation. Additionally, prolonged exposure to cariogenic infection in severe ECC cases may be associated with modulated immune responses, a phenomenon described in chronic inflammatory conditions. Nevertheless, the applicability of this mechanism to ECC remains to be clarified [22].

From a clinical perspective, CBC-derived indices such as NLR and SII offer practical, cost-effective, and readily available tools for assessing systemic inflammatory status. However, given the limited discriminative power observed in this study, these markers should be interpreted with caution and considered as supportive rather than definitive indicators in pediatric dental patients.

This study has several limitations. Its retrospective design limits causal inference, and the study population is restricted to children undergoing treatment under general anesthesia, which may affect generalizability. Additionally, the relatively small sample size of the low severity group (*n* = 11) may have reduced the statistical power of comparisons involving this subgroup and should therefore be considered when interpreting the findings. Therefore, the findings of the present study should be interpreted as associative rather than causal, and prospective longitudinal studies are needed to clarify the temporal relationship between ECC severity and systemic inflammatory responses. Additionally, the absence of advanced inflammatory biomarkers such as cytokine profiles limits a more comprehensive evaluation of systemic inflammation. Future prospective studies incorporating lesion-specific indices and broader biomarker panels are needed to further elucidate the relationship between ECC and systemic inflammatory responses. Furthermore, the study population was limited to children undergoing dental treatment under general anesthesia, as standardized preoperative CBC data were routinely available only in this clinical setting. Since hematological analyses are not routinely performed in children treated without general anesthesia, comparable retrospective laboratory data could not be consistently obtained. Therefore, the generalizability of the findings to the broader pediatric population may be limited.

## 5. Conclusions

The present study demonstrated that systemic inflammatory responses in early childhood caries do not increase proportionally with caries severity. Instead, a non-linear distribution pattern was observed, with relatively higher inflammatory activity in the moderate dmft group. Importantly, this study was conducted in a well-defined pediatric population undergoing dental treatment under general anesthesia, in which standardized preoperative hematological data are routinely available. This clinical setting provides a unique opportunity to evaluate systemic inflammatory responses in relation to oral disease under controlled conditions.

These findings suggest that systemic inflammation in ECC is influenced not only by caries burden but also by disease-related characteristics beyond numerical severity. The observed inflammatory alterations were mainly reflected in leukocyte-related parameters and immune-inflammatory indices, whereas hemoglobin and RDW values did not show significant differences among the groups. The availability of routinely collected hematological data in such clinical settings may facilitate the integration of systemic parameters into pediatric dental assessments without additional patient burden. Future studies conducted in broader and more heterogeneous populations, as well as those incorporating longitudinal or pre- and postoperative comparisons, are needed to further clarify the dynamics of systemic inflammatory responses in ECC.

## Figures and Tables

**Table 1:** Comparison of demographic parameters across dmft severity groups.

Demographic Parameters	Low Severity (*n* = 11)	Medium Severity (*n* = 92)	High Severity (*n* = 56)	*p* Value			
				Low Severity/Medium Severity	Low Severity/High Severity	Medium Severity/ High Severity	*R^2^*
Age (years) *	**3.91 ± 2.07**	**4.78 ± 1.41**	4.02 ± 1.26	0.069	0.816	**0.001**	0.071
	(2.52–5.30)	(4.49–5.08)	(3.68–4.36)				
Gender (F/M) ^υ^	3/8	47/45	28/28	0.203	0.202	0.999	
Height (cm)	102.7 ± 16.48	112.6 ± 11.72	106.5 ± 10.95	**0.026**	0.394	**0.010**	0.081
	(90.00–115.3)	(109.7–115.4)	(103.0–110.1)				
Weight (kg)	19.05 ± 9.74	19.61 ± 4.38	17.15 ± 3.78	0.744	0.280	**0.000**	0.059
	(12.08–26.02)	(18.67–20.55)	(16.12–18.17)				

* Values are presented as Mean ± SD (95% CI). Statistical analysis performed using one-way ANOVA. ^υ^ Categorical variables are presented as numbers. Statistical analysis was performed using the chi-square test. Bolded values indicate statistically significant differences (*p* < 0.05).

**Table 2:** Comparison of hematological and inflammatory parameters across dmft severity groups.

Hematological Parameters	Low Severity (*n* = 11)	Medium Severity (*n* = 92)	High Severity (*n* = 56)	*p* Value			
				Low Severity/Medium Severity	Low Severity/High Severity	Medium Severity/High Severity	*R* ^2^
NEUT %	**37.54 ± 14.12**	**45.42 ± 10.56**	39.53 ± 11.69	**0.026**	0.622	**0.002**	0.072
	(28.05–47.02)	(43.22–47.62)	(36.30–42.75)				
NEU# (×10^3^/µL)	3.45 ± 1.72	4.19 ± 2.96	3.29 ± 1.39	0.420	0.746	0.039	0.030
	(2.30–4.60)	(3.56–4.82)	(2.91–3.67)				
LYM %	51.99 ± 13.81	43.76 ± 11.16	50.75 ± 11.36	**0.027**	0.751	**0.000**	0.091
	(42.71–61.27)	(41.42–46.09)	(47.71–53.80)				
LYM# (×10^3^/µL)	4.65 ± 1.47	3.66 ± 1.29	4.14 ± 1.33	**0.020**	0.253	**0.036**	0.052
	(3.67–5.64)	(3.39–3.93)	(3.78–4.94)				
PLT (×10^3^/µL)	374.5 ± 47.71	353.2 ± 87.90	367.7 ± 90.44	0.434	0.811	0.338	0.008
	(342.4–406.5)	(335.0–371.4)	(343.5–391.9)				
RDW_CV (%)	13.26 ± 0.75	13.53 ± 1.23	13.61 ± 1.10	0.489	0.331	0.713	0.006
	(12.76–13.77)	(13.26–13.80)	(13.30–13.91)				
RDW_SD (fL)	39.50 ± 2.21	37.98 ± 5.18	39.04 ± 3.23	0.619	0.814	0.363	0.016
	(34.01–44.99)	(36.28–39.68)	(37.71–40.37)				
Hb (g/dL)	12.81 ± 0.99	12.81 ± 0.949	12.72 ± 0.836	0.995	0.762	0.573	0.002
	(12.14–13.47)	(12.61–13.01)	(12.49–12.95)				
NLR	0.88 ± 0.70	1.33 ± 1.32	0.89 ± 0.48	0.273	0.958	**0.021**	0.040
	(0.41–1.35)	(1.05–1.60)	(0.76–1.02)				
PLR	88.84 ± 34.56	112.7 ± 68.81	94.76 ± 27.81	0.262	0.538	0.068	0.028
	(65.65–112.1)	(98.36–127.0)	(87.25–102.3)				
SII	346.1 ± 326.3	475.9 ± 482.3	322.1 ± 190.9	0.388	0.742	**0.028**	0.034
	(126.9–565.3)	(375.4–576.3)	(269.5–374.7)				

NEUT %: Neutrophil percentage; NEU# (×10^3^/µL): Absolute neutrophil count; LYM %: Lymphocyte percentage; LYM# (×10^3^/µL): Absolute lymphocyte count; PLT (×10^3^/µL): Platelet count; RDW_CV (%): Red cell distribution width—coefficient of variation; RDW_SD (fL): Red cell distribution width—standard deviation; Hb (g/dL): Hemoglobin; NLR: Neutrophil-to-lymphocyte ratio; PLR: Platelet-to-lymphocyte ratio; SII: Systemic immune-inflammation index. Values are presented as Mean ± SD (95% CI). Statistical analysis performed using one-way ANOVA. Bolded values indicate statistically significant differences (*p* < 0.05).

## Data Availability

All data generated or analyzed during this study are included in this published article.

## Abbreviations

The following abbreviations are used in this manuscript:

ECC	Early Childhood Caries
CBC	Complete Blood Count
NLR	Neutrophil-to-Lymphocyte Ratio
PLR	Platelet-to-Lymphocyte Ratio
SII	Systemic Immune-Inflammation Index
dmft	Decayed, Missing, and Filled Teeth (primary dentition)
NEU	Neutrophil
LYM	Lymphocyte
PLT	Platelet
RDW-CV	Red Cell Distribution Width—Coefficient of Variation
RDW-SD	Red Cell Distribution Width—Standard Deviation
Hb	Hemoglobin
GA	General Anesthesia
ASA	American Society of Anesthesiologists
